# Adaptation to climate change: trade‐offs among responses to multiple stressors in an intertidal crustacean

**DOI:** 10.1111/eva.12394

**Published:** 2016-06-30

**Authors:** Morgan W. Kelly, Melissa B. DeBiasse, Vidal A. Villela, Hope L. Roberts, Colleen F. Cecola

**Affiliations:** ^1^ Department of Biological SciencesLouisiana State UniversityBaton RougeLAUSA

**Keywords:** adaptation, climate change, comparative physiology, contemporary evolution, ecological genetics, experimental evolution, transcriptomics

## Abstract

Trade‐offs may influence both physiological and evolutionary responses to co‐occurring stressors, but their effects on both plastic and adaptive responses to climate change are poorly understood. To test for genetic and physiological trade‐offs incurred in tolerating multiple stressors, we hybridized two populations of the intertidal copepod *Tigriopus californicus* that were divergent for both heat and salinity tolerance. Starting in the F_2_ generation, we selected for increased tolerance of heat, low salinity, and high salinity in replicate lines. After five generations of selection, heat‐selected lines had greater heat tolerance but lower fecundity, indicating an energetic cost to tolerance. Lines selected for increased salinity tolerance did not show evidence of adaptation to their respective environments; however, hypo‐osmotic selection lines showed substantial loss of tolerance to hyperosmotic stress. Neither of the salinity selection regimes resulted in diminished heat tolerance at ambient salinity; however, simultaneous exposure to heat and hypo‐osmotic stress led to decreased heat tolerance, implying a physiological trade‐off in tolerance to the two stressors. When we quantified the transcriptomic response to heat and salinity stress via RNA sequencing, we observed little overlap in the stress responses, suggesting the observed synergistic effects of heat and salinity stress were driven by competing energetic demands, rather than shared stress response pathways.

## Introduction

In many ecosystems, the environment will change rapidly along multiple axes in the coming century, so that the physiological demands imposed by multiple stressors may constrain both plastic and evolutionary responses to global change (Crain et al. 2008; Hoffmann and Sgrò 2011; Boyd et al. 2015; Deutsch et al. 2015). These constraints will be driven by trade‐offs, which occur whenever two traits cannot be simultaneously optimized (Agrawal et al. 2010). This can happen at a genetic level, when two traits are genetically correlated, thereby constraining evolution if selection acts in a direction that opposes the direction of their correlation (Walsh and Blows 2009; Bubliy et al. 2012). Trade‐offs can also occur at the level of an individual organism's physiology, when resources allocated to one function cannot be allocated to another, thereby constraining the ability perform two functions (e.g., tolerate co‐occurring stressors) simultaneously (Sokolova 2013).

Genetic trade‐offs occur because genetic variation and natural selection are usually multivariate, and evolution depends on the degree to which genetic variation is aligned with the ‘multivariate direction of selection’ (Blows and Hoffmann 2005). Although genetic correlations are unlikely to completely halt evolution (Schluter 1996), they can easily slow the rate of adaptation (Walsh and Blows 2009), an important consideration given the present rate of environmental change (Hellmann and Pinedakrch 2007; Lau and terHorst 2015). For example, a population might possess genetic variation for tolerance of high salinity, and genetic variation for tolerance of thermal stress, but if there is a negative correlation between these two traits, adaptation to both stressors simultaneously will occur more slowly than to either one in isolation, such that the diminished rate of evolutionary change might preclude evolutionary rescue. Relatively few studies have considered trade‐offs involved in stress adaptation (but see Etterson and Shaw 2001; Williams et al. 2012; Oakley et al. 2014; Chirgwin et al. 2015; Sørensen et al. 2015), limiting the ability to predict both the rate and correlated effects of evolutionary responses to environmental change.

Physiological trade‐offs typically occur through competing demands on metabolism (Sokolova 2013). For example, hypo‐osmotic stress can increase energy requirements of homeostasis through energetic costs of ion regulation (Kidder et al. 2006), and in ectotherms, the energetic requirements of basal metabolism increase with increasing ambient temperature. Thus, if available energy remains constant, higher temperatures should lower tolerance of hypo‐osmotic stress through competing demands for the same pool of resources. In the absence of energetic constraints, it is also possible for two functions to trade‐off more directly, as in the case where two functions require opposing changes in gene regulation. In either case, the effects of multiple stressors are poorly understood (Altshuler et al. 2011), and because stressors may interact in nonadditive ways, the potential effects of multiple stressors often cannot be understood from single‐stressor studies.

Importantly, two traits may be positively genetically correlated, but nevertheless experience a physiological trade‐off. For example, if stress tolerance traits are limited by resource availability, genotypes with greater ability to acquire resources may exhibit greater tolerance to both heat and hypo‐osmotic stress, leading the two traits to appear to be positively genetically correlated (Houle 1991). Nevertheless, the competing energy demands imposed by each stressor may mean that *simultaneous* exposure to hypo‐osmotic stress would lower heat tolerance.

Either physiological or genetic trade‐offs may alter the demographic response to environmental change. Within a generation, synergistic physiological effects of multiple stressors may produce a greater demographic impact than would have been predicted from either of the stressors’ individual effects. Across multiple generations, negative genetic correlations among stress tolerance traits may alter the likelihood of evolutionary rescue if selection for one tolerance trait tends to deplete genetic variation for the response to other stressors. However, despite their importance, the possibility of both physiological and genetic trade‐offs have rarely been tested together in the same system.

Here, we report the results of a series of experiments using the intertidal copepod, *Tigriopus californicus,* to test responses to two stressors commonly faced by organisms in coastal habitats: heat stress and changes in salinity. *T. californicus* is a small (~1 mm) crustacean, abundant in rocky splash pools along the Pacific coast of North America. These pools are not regularly inundated by tides, and experience large fluctuations in both temperature and salinity. As a consequence, *Tigriopus* is extremely tolerant of fluctuations in both temperature (Kelly et al. 2012) and salinity, with the ability to survive salinities ranging from 2 to 100 ppt (<1/10th to 3× normal sea water; Burton and Feldman 1983). Heat and high salinity stress often co‐occur in this habitat, with pools becoming saltier and more thermally stressful as they heat up and evaporate. Shallower pools also experience greater hypo‐osmotic stress, as they are more easily flushed by rainwater. Given that heat and salinity stress may co‐vary in space and in time, we were interested in whether responses to these stressors might incur trade‐offs with responses to the others.

We tested for trade‐offs on both physiological and evolutionary timescales: We tested whether the evolution of increased tolerance of one stressor would lead to a correlated loss in tolerance of the others, and we tested whether simultaneous exposure to these stressors had synergistic physiological effects. To test for evolutionary trade‐offs, we hybridized two populations that were divergent for both heat and salinity tolerance, selected for increased tolerance of either heat, hyperosmotic, or hypo‐osmotic stress in replicate hybrid lines, and then tested whether evolution of increased tolerance to one stressor led to loss of tolerance to one of the others. Selection experiments are one of the most powerful tests for ecological tradeoffs, allowing genetic correlations among traits to be disentangled from correlated sources of selection (Fry 2003; Berger et al. 2014). In many laboratory selection experiments, the response to selection depends on standing genetic variation in the source population or new mutations that occur over the time frame of the experiment. By hybridizing populations that were divergent for heat and salinity stress and then selecting for increased tolerance in hybrids, we were able to directly target the alleles responsible for phenotypic divergence between these populations, separated by millions of generations of evolution, and ask whether the alleles responsible for differences in heat or salinity tolerance between the two populations have pleiotropic effects on tolerance of other stressors. We observed physiological, but not genetic trade‐offs to tolerance of multiple stressors. Our results have important implications for biological responses to environmental change, as they imply that the synergistic effects of multiple simultaneous stressors may impose a more important demographic constraint than trade‐offs imposed by negative genetic correlations among stress tolerance traits.

## Methods

### Field collection, crosses, and selection

Field collection and copepod culture are described in detail in Kelly et al. (2012). Briefly, we established laboratory cultures of *T. californicus* from two sites, Bodega Bay in northern California, USA (BB 39°20′N, 123°33′W) and San Diego in southern California (SD, 32°49′N, 117°16′W). At each site, we collected individuals from 3 to 4 tidepools. We initiated one laboratory culture for each pool, with 50 gravid females per culture, and maintained cultures at 19°C (*Tigriopus* pools in nature range from 0 to 35°C) under 12‐h light/12‐h dark conditions at ambient (35 ppt) salinity. We maintained cultures in the laboratory for two generations before establishing crosses. We kept generations separate and established each new generation with 40 haphazardly selected mate‐guarding pairs.

Previous work has established that copepods from northern and southern sites differ in their thermal tolerances, with LT_50_ temperatures of 34.8 and 36.5°C for BB and SD, respectively, and also that genetic variation for thermal tolerance within sites is quite limited, with 99% of genetic variation for thermal tolerance partitioned between sites (Kelly et al. 2012). We initiated the cross with 20 males from SD and 20 females from BB, teasing apart mate‐guarding pairs (males + virgin females) with a fine probe and then pairing each individual with a partner from the opposite population. To ensure successful mating had occurred, each pair was held separately in a 24‐well tissue culture plate until the female had produced her first brood. After that, all females and broods were combined into a single 500‐mL culture, and maintained as described above. We could be sure that each female mated only with the intended male because female *Tigriopus* mate once (after the final molt) and produce all subsequent broods from stored sperm (Burton 1985). Given that most of the genetic variation for thermal tolerance is partitioned between populations, we expected 20 individuals from each population to be sufficient to sample the majority of the genetic variation for thermal tolerance segregating between populations.

**Figure 1:**
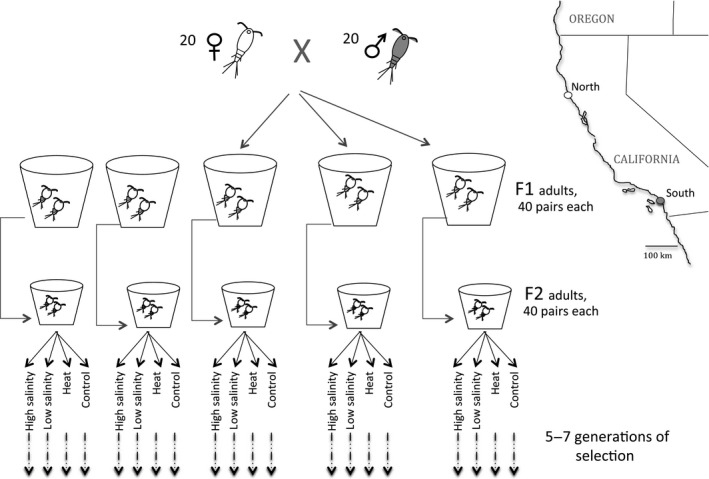
Study design: Experimental crosses plus selection to test for genetic trade‐offs incurred in adapting to heat and salinity stress in the copepod *Tigriopus californicus*.

When the F1 generation reached adulthood, we created five replicate cultures, each established with 30 mate‐guarding pairs from the F1 generation (Fig. 1). Replicate cultures were also maintained at 19°C as described above. Starting in the F2 generation, we split each replicate line into three selection treatments, selecting for tolerance to heat, low salinity, and high salinity, respectively. Selection lines were propagated for five more generations, before remeasuring heat tolerance and testing for trade‐offs between heat and salinity tolerance. From each F2 line, we also established an ‘unselected’ control with 40 haphazardly chosen pairs each generation.

To select for increased heat tolerance, we exposed >80 mate‐guarding pairs to the temperature that produced 50–90% mortality for 1 h (36.0°C in the first generation of selection, increasing by ~0.1°C per generation of selection). We then established the next generation with 30 of the surviving mate‐guarding pairs. To select for increased tolerance of low salinity and high salinity, we placed 50 mate‐guarding pairs at 20 and 55 ppt, respectively, for each of five replicate selection lines. Thereafter, we decreased salinity by 2 ppt per generation for the low‐salinity selection lines, and increased it by 3 ppt per generation for the high‐salinity selection lines, and established each new generation with 50 mate‐guarding pairs.

### Measurement of heat tolerance

After five generations of selection, we measured the effect of selection for increased heat tolerance on upper lethal limits following Kelly et al. (2012). For each of the heat‐selected lines and unselected controls, we exposed sets of nine mate‐guarding pairs to a target temperature for 1 h, allowed 48 h for recovery, and then assessed survival. We did this for a series of 5–10 temperatures at 0.2°C intervals, spanning from the temperature that produced 90–100% survival to the temperature that produced 90–100% mortality, and then used the mortality at each temperature to estimate LT_50_ for each line via logistic regression in the statistical program R (R Development Core Team 2014). For each of the high‐ and low‐salinity selection lines, we measured heat tolerance twice: once for individuals that had been taken from the salinity selection line after five generations of selection, and then held under common garden conditions at ambient (35 ppt) salinity for one generation. Second, we measured heat tolerance for individuals from each selection line at their ‘native’ salinity (70 ppt for high‐salinity lines, 15 ppt for low‐salinity lines) after six generations of selection. This allowed us to separate any evolutionary loss of heat tolerance that might have occurred in the salinity selection lines from the possible synergistic physiological effects of simultaneously tolerating both heat and salinity stress. After estimating LT_50_ for each line, we tested for differences in temperature tolerance among treatments in two‐way ANOVAs, with LT_50_ estimates for each line × treatment combination as the response variable (R Development Core Team 2014). The first ANOVA tested for effects of heat selection and sex on heat tolerance at generation five of selection. The second ANOVA tested for effects of selection + salinity treatment and sex on heat tolerance at generation six of selection. We performed two separate ANOVAs for this analysis because each experimental test was paired with its relevant unselected control for that generation (five generations of selection for heat tolerance and 6 generations of selection for salinity) to account for minor fluctuations in incubator conditions and food quality, which might affect heat tolerance.

### Measurement of fecundity

To test for an effect of selection treatment and ambient salinity on fecundity, we placed mate‐guarding pairs from each of the selection lines and controls (*N* = 6–8 per line × salinity treatment) in 15‐mL six‐well plates filled with artificial seawater at one of three test salinities: 20 ppt (low), 35 ppt (ambient), and 50 ppt (high) and maintained them at 19°C under 12‐h L: 12‐h D conditions. All adults and larvae in this experiment were fed ground spirulina fish food *ad libitum*. We followed each female until she produced three broods, or for 21 days, whichever came first. We recorded the day the female of each pair first became gravid and the dates of her first three broods. Each time a new brood hatched, the female was moved to a new well in the plate. Offspring from each brood were counted by individually pipetting larvae to a new dish. We calculated larvae produced per day for each female as the total number of larvae in the first three broods, divided by the number of days required to produce the first three broods, from the day she first became gravid. For females that produced fewer than three broods, we divided the total number of offspring produced in 21 days by 21. About 20% of females in each treatment failed to produce any larvae. We expected some level of brood failure, as crosses among populations of *T. californicus* experience substantial outbreeding depression (Edmands 1999), and even seven generations after the initial cross, these lines have likely not been completely purged incompatibilities leading to reproductive failure. A logistic regression indicated no differences between selection treatments or rearing salinities in the probability of brood failure, so females producing zero larvae were removed from further analysis. We tested for an effect of selection treatment and rearing salinity on larvae produced per day in a two‐way ANOVA in the statistical program R, with individual selection lines + salinity treatments as replicates.

### Measurement of salinity tolerance and maintenance costs

We tested for an effect of selection treatment and ambient salinity on maintenance costs using a starvation experiment, under the assumption that selection + salinity treatment combinations with the lowest maintenance costs would have the longest survival during starvation (Evers and Kooijman 1988). Three groups of ten adult females from each selection line were placed under common garden acclimation conditions (35 ppt, 19°C) for 72 h, and then one group of ten was moved to each of three test salinities: low (5 ppt), medium (35 ppt), or high (90 ppt), and held without food at 19°C for 17 days. We checked each container for mortality every 2–3 days and estimated per‐day mortality rates via individual cox proportional hazard models, fitted to each selection line × salinity combination in the R package ‘OIsurvival.’ We tested for effects of salinity, selection regime, and a selection × salinity interaction in a two‐way ANOVA, with individual lines as replicates, after first log‐transforming per‐day mortality values to achieve normality.

### Transcriptome sequencing

We measured the transcriptomic response to heat‐shock and to hypo‐ and hyperosmotic stress using RNA sequencing. The response to heat shock was measured as part of a prior experiment (Kelly et al. 2016). Briefly, we created six sequencing libraries, each from the pooled RNA of 30 hybrid copepods from the F4 generation. We created two libraries for each of three unselected control lines: one from 30 adults (males and females) that had been held at ambient temperatures and then flash‐frozen, and one from copepods that had been heat‐shocked for 1 h at 34°C, allowed 1 h to recover, and then flash‐frozen. To measure the transcriptomic response to low and high salinities, we created three libraries from individuals from the SD population for each of three salinities [low (15 ppt), ambient (35 ppt), and high (60 ppt)]: 50 copepods in each replicate were exposed to each salinity treatment for 1 h before flash‐freezing in liquid nitrogen.

Total RNA for all treatments was immediately extracted using the Qiagen RNeasy Plus Mini Kit. mRNA was isolated using the NEBNext Poly(A) mRNA Magnetic Isolation kit and libraries were prepared using either the NEBNext Ultra RNA library prep kit for Illumina and the AxyPrep Mag PCR Clean‐up kit (salinity experiment) or the Illumina TruSeq kit (heat‐shock experiment) (version 2; Illumina, San Diego, CA, USA), following the manufacturer's instructions. The distribution of fragment sizes was verified by running libraries on a Bioanalyzer high‐sensitivity DNA chip, and library concentration was quantified with qPCR using a Kapa Biosystems kit. Barcoded libraries were pooled in equimolar concentrations. 100‐bp single‐end reads were sequenced in two lanes (9–12 libraries per lane) on the HiSeq2500 platform using a TruSeq SBS sequencing kit v4 at the University of Illinois at Urbana‐Champaign Roy J. Carver Biotechnology Center (salinity experiment) or at the University of Davis Genome Center Core Facility (heat‐shock experiment).

### Quality control and gene expression estimation

Reads were trimmed of adapter sequences and low‐quality bases using the wrapper script Trim Galore v3.8 with Cutadapt v1.7 and FastQC v0.11.2 under default settings. We mapped reads to a transcriptome previously assembled from reads for the SD population by Kelly et al. (accepted). Briefly, the final transcriptome was assembled from 44,617,800 pairs of reads to obtain a transcriptome sequence for the southern population. The final collapsed and filtered assembly contained 59,519 contigs with an N_50_ of 2969 bp and a GC content of 47.3%.

To measure gene expression, reads from each sequencing library were mapped to the transcriptome assembly separately using the default settings of RSEM v. 1.2.7 (Li and Dewey 2011). We investigated patterns of gene expression using the R Bioconductor package, limma (Law et al. 2014; Ritchie et al. 2015). Limma employs an empirical Bayesian method to estimate log‐fold changes in expression and has recently been shown to be more robust to false positives than methods that rely heavily on fitting a negative binomial distribution to the data (Law et al. 2014). In limma, we fit a generalized linear model to the data, testing for differences in gene expression between heat‐shocked and ambient temperature treatments, between low‐ and ambient‐salinity treatments, and between high‐ and ambient‐salinity treatments, setting the threshold for false discovery in all analyses to FDR < 0.05 (Benjamini and Hochberg 1995).

## Results

### Heat tolerance

**Figure 2:**
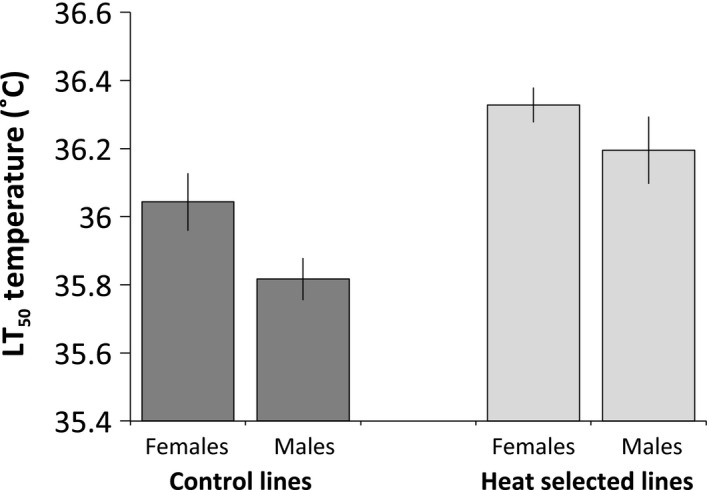
Median lethal temperatures (LT_50_) for male and female *Tigriopus californicus* from hybrid lines selected for increased heat tolerance for five generations, and for unselected controls. LT_50_ temperatures for each of five replicates per treatment were estimated via logistic regression.

After five generations of selection for increased heat tolerance, we measured the effect of selection on upper lethal limits following Kelly et al. (2012) and estimated median lethal temperatures (LT_50_) temperatures for each line via logistic regression. A two‐way ANOVA revealed differences in LT_50_ temperatures as a function of sex (*F*_1,16_ = 6.9, *P* = 0.02) and selection treatment (*F*_1,16_ = 23.6, *P* = 0.0002), with heat‐selected lines exhibiting ~0.3°C greater heat tolerance in females, and ~0.5°C greater heat tolerance in males, in comparison with unselected controls (Fig. 2).

**Figure 3:**
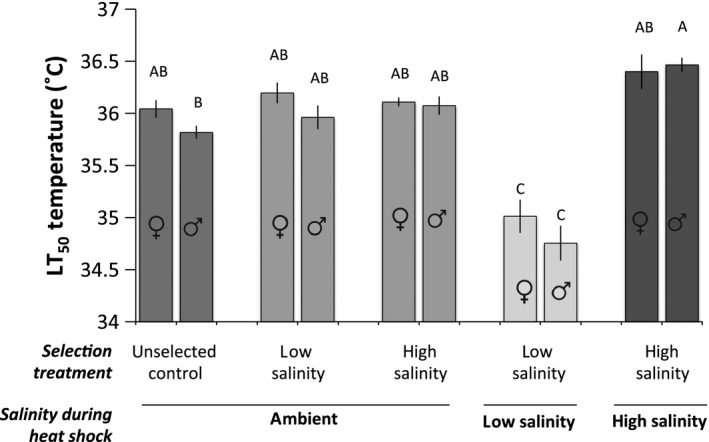
Median lethal temperatures (LT_50_) for male and female *Tigriopus californicus* from hybrid lines selected for increased low and high salinity tolerance for five generations, and then either returned to ambient salinity, or held at their respective salinities for a sixth generation of selection. LT_50_ temperatures for each of four replicate lines per salinity/selection combination were estimated via logistic regression. Shared letters above bars indicate treatments whose means do not differ (Tukey–Kramer, *P* > 0.05).

After six generations of selection, we tested for an effect both of adaptation to salinity stress and of simultaneous heat and salinity stress on heat tolerance. A two‐way ANOVA revealed differences in LT_50_ temperatures among treatments (*F*_9,36_ = 28.7, *P* < 0.0001, Fig. 3). However, a *post hoc* test revealed no differences in heat tolerance between selected lines and unselected controls when all lines were held under common (35 ppt) conditions (Tukey–Kramer, *P* > 0.05). Relative to controls at ambient salinity, there was a ~1.0°C reduction in heat tolerance when low‐salinity selected lines were held at low salinity (15 ppt), and an increase in heat tolerance in high‐salinity selected males, when held at high salinity (70 ppt).

### Fecundity and maintenance costs

**Figure 4:**
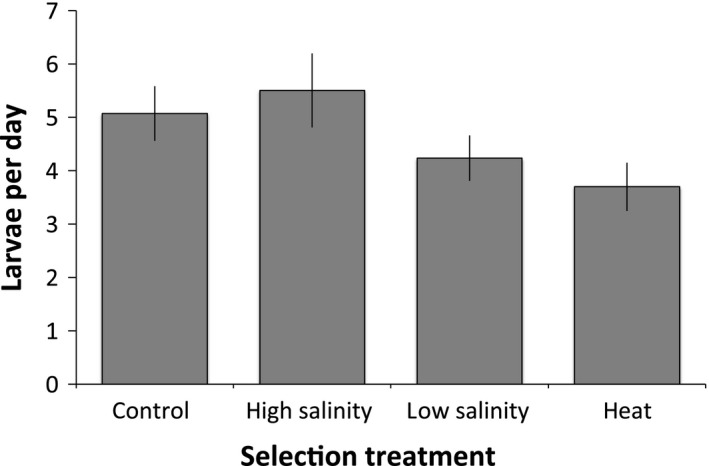
Mean fecundity (larvae per day, ±SE) for female *Tigriopus californicus* from hybrid lines selected for five generations for increased tolerance of heat, high salinity stress, and low salinity stress, as well as for unselected control lines.

**Figure 5:**
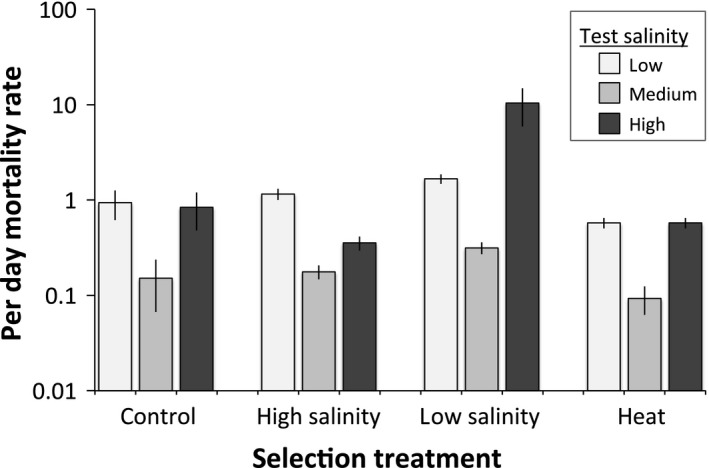
Per‐day mortality rates for adult female *Tigriopus californicus* from four different selection treatments at low (5 ppt), medium (35 ppt), or high (90 ppt) salinities. Bars show mortality rate (estimated via cox proportional hazard model), mean ± SE.

A two‐way ANOVA revealed no effect of salinity on fecundity (*F*_2,35_ = 2.39, *P* = 0.11), but a marginally significant effect of selection (*F*_3,35_ = 2.86, *P* = 0.051) with the lowest fecundity in heat‐selected lines (Fig. 4). After eight generations of selection, we used a starvation experiment to test for an effect of selection regime and ambient salinity on maintenance costs. A two‐way ANOVA revealed an effect of selection regime (*F*_3,36_ = 9.96, *P* < 0.0001), and salinity stress (*F*_2,36_ = 32.1, *P* < 0.0001) on mortality rate, as well as an interaction between the two effects (F_6,36_ = 2.38, *P* = 0.048, Fig. 5). However, a *post hoc* test provided no evidence for adaptation of either the low‐ or high‐salinity selection lines to their native salinity regimes, as neither had a mortality rate lower than the control at its respective salinity (Tukey HSD > 0.05). We observed the highest mortality rate in hypo‐osmotic selection lines under high salinity stress, which had a greater mortality rate than all of the other selection and salinity treatment combinations in a *post hoc* comparison (Tukey HSD, *P* < 0.05).

### Gene expression

**Figure 6:**
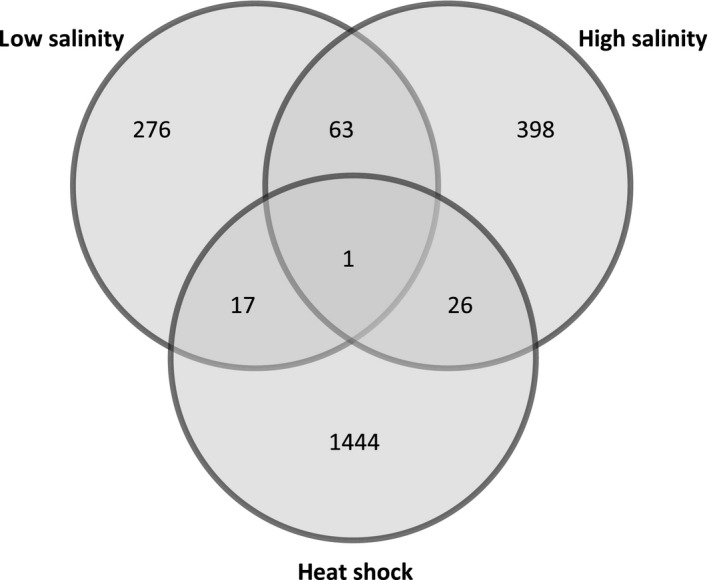
Venn diagram showing number of differentially expressed transcripts in *Tigriopus californicus* in response to heat shock (1 h at 34°C), low salinity stress (1 h at 15 ppt salinity), and high salinity stress (1 h at 60 ppt salinity), FDR < 0.05.

We mapped sequence reads to a previously assembled reference transcriptome and fit a generalized linear model to the data, testing for differences in gene expression between heat‐shocked and ambient temperature treatments, between low‐ and ambient‐salinity treatments, and between high‐ and ambient‐salinity treatments. Setting the threshold for false discovery in all analyses to FDR < 0.05 (Benjamini and Hochberg 1995), we identified 1488, 357, and 488 transcripts responding to heat shock, low salinity, and high salinity, respectively, but very little overlap among the transcripts responding to each stressor (Fig. 6). The transcriptional response to heat shock was enriched for catalytic, hydrolase, and exopeptidase activities, while the hyperosmotic response was enriched for protein binding, response to chemical, and protein homodimerization activity (Fisher's exact test, *P* < 0.05). The functional category anion transmembrane transporter activity was significantly enriched in the response to low salinity (Fisher's exact test, *P* < 0.05).

## Discussion

Trade‐offs may influence both physiological and evolutionary responses to co‐occurring stressors, but their potential importance in shaping plastic and adaptive responses to climate change is poorly understood. We used laboratory selection and physiological tolerance experiments to test whether responses to heat and salinity stress (two important stressors in intertidal habitats) would incur trade‐offs in the copepod *T*. *californicus*. When we crossed populations that were divergent for heat and salinity tolerance and then selected for increased heat tolerance for five generations, we observed increased heat tolerance in selected lines, but also reduced fecundity. This finding is in contrast to previous work, where we were unable to demonstrate a fecundity cost in female *Tigriopus* selected for increased heat tolerance (Kelly et al. 2013). However, in these previous experiments, the response to selection was driven by genetic variation *within* populations; it possible that alleles responsible for variation in heat tolerance *between* populations carry a larger cost (Willett 2010).

We also saw no evidence that selection for increased salinity tolerance leads to a loss of heat tolerance, indicating that the two traits are not genetically correlated. We selected for increased tolerance of both hyper‐ and hypo‐osmotic stress in hybrids by serially increasing or decreasing the rearing salinity of replicate lines over the course of five generations, but when we returned copepods from selected lines to ambient (35 ppt salinity), we observed no difference in heat tolerance of selected lines and controls. Our results are consistent with previous work in *Daphnia* where lines selected for increased salinity tolerance also failed to show a cost of increased salinity tolerance at nonstressful salinities (Latta et al. 2012). We also saw no evidence that selection for increased heat tolerance lead to decreased salinity tolerance, with similar survival at both high and low salinities for heat‐selected lines and controls.

In contrast to results for the heat tolerance selection lines, we saw no evidence that the salinity selection lines had adapted to their native salinity regimes, with neither the low‐ or high‐salinity lines having improved fecundity or survival at their native salinities. However, despite the lack of adaptation to their native salinity, the low‐salinity lines showed substantial loss of tolerance to high salinity, with nearly 10‐fold greater mortality than controls at high salinity. One possible reason for lack of adaptation in the salinity selection lines is that we did not impose strong enough selection. In the heat selection lines, we imposed at least 50% mortality every generation, whereas the salinity selection lines just experienced a gradual increase or decrease in salinity. Alternately, it is possible that there is simply less additive genetic variance for salinity tolerance segregating between populations than there is for heat tolerance.

An important challenge in interpreting our results is the extensive hybrid breakdown observed in interpopulation crosses of *Tigriopus* (Edmands 1999; Burton 1990; Ellison and Burton 2008). Hybrids typically show a dramatic decline in fitness in the F_2_ generation, followed by generations of interlocus selection that purges incompatible alleles, so that hybrid populations may eventually exceed the fitness of either parental population (Pereira et al. 2014). This underscores the importance of comparing selected lines with hybrid controls from the same cross, at the same generation. Because we expect the average effects of post‐hybridization intralocus selection to be the same for both selected lines and controls, we can then attribute changes in tolerance or fecundity (relative to the hybrid controls) to the effects of the selection regime, rather to the side effects of hybrid breakdown.

Despite the apparent lack of genetic correlation between heat and salinity tolerance, we observed synergistic physiological effects of heat and hypo‐osmotic stress, with copepods from low‐salinity lines held at their ‘native’ (15 ppt) salinity having ~1°C lower heat tolerance than control lines at ambient (35 ppt) salinity. However, we saw no evidence that the apparent physiological trade‐off between heat and salinity tolerance was driven by opposing effects on gene regulation. We observed 1488 transcripts that were differentially regulated in response to heat shock, but only 18 overlapped with the 357 transcripts responding to low salinity, indicating that the two stressors trigger unrelated physiological pathways. More likely, the observed synergistic effects of the two stressors are driven by competing energetic demands imposed by tolerating the two stressors. The response to hypo‐osmotic stress was dominated by transcripts involved in ion regulation, which may be quite costly (Kidder et al. 2006). In contrast, *Tigriopus* respond to heat shock through a combination of protein stabilization, and hydrolysis of mis‐folded aggregated proteins that accumulate after heat shock (Schoville et al. 2012). Although they involve nonoverlapping pathways, each response might incur energetic demands that would reduce tolerance to the other stressor. In addition, increased temperatures lead to increased permeability of membranes (Hochachka and Somero 1984), and so are likely to decrease the efficacy of active ion regulation.

Interestingly, exposure to hyperosmotic stress led to slightly higher heat tolerance (at least in males). This was not reflected the level of overlap among differentially expressed genes: Only 27 differentially expressed genes were shared between the two stress responses. However, the positive effects of hyperosmotic stress on heat tolerance might still be driven by a *functional* overlap in the two stress responses. The response to hyperosmotic stress was dominated by transcripts involved in protein stabilization, also a major component of the heat‐shock response (Feder and Hofmann 1999; Schoville et al. 2012). In particular, ten of the transcripts that were upregulated in response to hyperosmotic stress were heat‐shock proteins, mostly isoforms of hsp 70. Although this was small component of the total response, recent work using RNAi to manipulate gene expression has shown that a change in the expression of a single heat‐shock protein can alter heat tolerance (Barreto et al. 2014).

The interactive effects of co‐occurring stressors will depend on the specific biology of individual stress responses. Here, we have shown that, depending on the physiological basis of the two responses, they can either dampen each other's effects, as in the case of heat and hyperosmotic stress, or heighten each other's effects, as in the case of heat and hypo‐osmotic stress. Our results highlight the need for more physiological studies in the context of responses to multiple stressors, as the responses we observed depended on the specific combination of stressors, and could not have been predicted from the individual stress responses. Our results also highlight the utility of transcriptomic techniques for understanding physiological responses to multiple stressors: relatively few studies employed transcriptomic techniques in this context (DeBiasse and Kelly 2015), and the upregulation of heat‐shock proteins under hyperosmotic stress provided a mechanism for the positive effect of hyperosmotic stress on heat tolerance, a result that would otherwise have gone unexplained. Finally, our results also have important implications for biological responses to environmental change. As one of the few studies to test both physiological and genetic trade‐offs incurred in tolerating multiple stressors, our findings imply that the synergistic physiological effects of multiple stressors may have more important consequences than evolutionary constraints imposed by genetic correlations among stress tolerance traits.

## Data accessibility

Data from this manuscript are available from the Dryad Digital Repository: http://dx.doi.org/10.5061/dryad.kj6j1.

## References

[eva12394-bib-0001] Agrawal, A. A. , J. K. Conner , and S. Rasmann 2010 Tradeoffs and negative correlations in evolutionary ecology In Bell M. A., Eanes W. F., Futuyma D. J., and Levinton J. S., eds. Evolution after Darwin: The First 150 Years, pp. 243–268. Sinauer Associates, Sunderland, MA.

[eva12394-bib-0002] Altshuler, I. , B. Demiri , X. Sen , A. Constantin , N. D. Yan , and M. E. Cristescu 2011 An integrated multi‐disciplinary approach for studying multiple stressors in freshwater ecosystems: *Daphnia* as a model organism. Integrative and Comparative Biology 51:623–633. 21873644 10.1093/icb/icr103

[eva12394-bib-0003] Barreto, F. S. , S. D. Schoville , and R. S. Burton 2014 Reverse genetics in the tide pool: knock‐down of target gene expression via RNA interference in the copepod *Tigriopus californicus* . Molecular Ecology Resources :1–12, doi: 10.1111/1755‐0998.12359. http://www.ncbi.nlm.nih.gov/pubmed/25487181. 25487181 10.1111/1755-0998.12359

[eva12394-bib-0004] Benjamini, Y. , and Y. Hochberg 1995 Controlling the false discovery rate: a practical and powerful approach to multiple testing. Journal of the Royal Statistical Society. Series B 57:289–300.

[eva12394-bib-0005] Berger, D. , R. J. Walters , and W. U. Blanckenhorn 2014 Experimental evolution for generalists and specialists reveals multivariate genetic constraints on thermal reaction norms. Journal of Evolutionary Biology 27:1975–1989. 25039963 10.1111/jeb.12452

[eva12394-bib-0006] Blows, M. W. , and A. A. Hoffmann 2005 A reassessment of genetic limits to evolutionary change. Ecology 86:1371–1384, Eco Soc America.

[eva12394-bib-0007] Boyd, P. W. , S. T. Lennartz , D. M. Glover , and S. C. Doney 2015 Biological ramifications of climate‐change‐mediated oceanic multi‐stressors. Nature Climate Change 5:71–79, Nature Publishing Group.

[eva12394-bib-0008] Bubliy, O. A. , T. N. Kristensen , V. Kellermann , and V. Loeschcke 2012 Plastic responses to four environmental stresses and cross‐resistance in a laboratory population of *Drosophila melanogaster* . Functional Ecology 26:245–253.

[eva12394-bib-0009] Burton, R. S. 1985 Mating system of the intertidal copepod *Tigriopus californicus* . Marine Biology 86:247–252.

[eva12394-bib-0010] Burton, R. S. 1990 Hybrid breakdown in developmental time in the copepod *Tigriopus californicus* . Evolution 44:1814–1822, JSTOR. 10.1111/j.1558-5646.1990.tb05252.x 28567806

[eva12394-bib-0011] Burton, R. S. , and M. W. Feldman 1983 Physiological effects of an allozyme polymorphism: glutamate‐pyruvate transaminase and response to hyperosmotic stress in the copepod *Tigriopus californicus* . Biochemical Genetics 21:239–251. 6860293 10.1007/BF00499136

[eva12394-bib-0012] Chirgwin, E. , K. Monro , C. M. Sgro , and D. J. Marshall 2015 Revealing hidden evolutionary capacity to cope with global change. Global Change Biology 21:3356–3366. 25781417 10.1111/gcb.12929

[eva12394-bib-0013] Crain, C. M. , K. Kroeker , and B. S. Halpern 2008 Interactive and cumulative effects of multiple human stressors in marine systems. Ecology Letters 11:1304–1315. 19046359 10.1111/j.1461-0248.2008.01253.x

[eva12394-bib-0014] DeBiasse, M. B. , and M. W. Kelly 2015 Plastic and evolved responses to global change: what can we learn from comparative transcriptomics? Journal of Heredity 107:71–81. 26519514 10.1093/jhered/esv073

[eva12394-bib-0015] Deutsch, C. , A. Ferrel , B. Seibel , H.‐O. Pörtner , and R. B. Huey 2015 Climate change tightens a metabolic constraint on marine habitats. Science 348:1132–1136. 26045435 10.1126/science.aaa1605

[eva12394-bib-0016] Edmands, S. 1999 Heterosis and outbreeding depression in interpopulation crosses spanning a wide range of divergence. Evolution 53:1757–1768. 10.1111/j.1558-5646.1999.tb04560.x 28565458

[eva12394-bib-0017] Ellison, C. K. , and R. S. Burton 2008 Interpopulation hybrid breakdown maps to the mitochondrial genome. Evolution 62:631–638, Wiley Online Library. 18081717 10.1111/j.1558-5646.2007.00305.x

[eva12394-bib-0018] Etterson, J. R. , and R. G. Shaw 2001 Constraint to adaptive evolution in response to global warming. Science 294:151–154. 11588260 10.1126/science.1063656

[eva12394-bib-0019] Evers, E. G. , and S. A. L. M. Kooijman 1988 Feeding, digestion and oxygen consumption in *Daphnia magna* a study in energy budgets. Netherlands Journal of Zoology 39:56–78, Brill.

[eva12394-bib-0020] Feder, M. E. , and G. E. Hofmann 1999 Heat‐shock proteins, molecular chaperones, and the stress response: evolutionary and ecological physiology. Annual Review of Physiology 61:243–282. 10.1146/annurev.physiol.61.1.243 10099689

[eva12394-bib-0021] Fry, J. D. 2003 Detecting ecological trade‐offs using selection experiments. Ecology 84:1672–1678.

[eva12394-bib-0022] Hellmann, J. , and M. Pinedakrch 2007 Constraints and reinforcement on adaptation under climate change: selection of genetically correlated traits. Biological Conservation 137:599–609.

[eva12394-bib-0023] Hochachka, P. W. , and G. N. Somero 1984 Biochemical Adaptation. Princeton University Press, Princeton, NJ.

[eva12394-bib-0024] Hoffmann, A. A. , and C. M. Sgrò 2011 Climate change and evolutionary adaptation. Nature 470:479–485. 21350480 10.1038/nature09670

[eva12394-bib-0222] Houle, D. 1991 Genetic covariance of fitness correlates: what genetic correlations are made of and why it matters. Evolution in Health and Disease 45:630‐648. 10.1111/j.1558-5646.1991.tb04334.x 28568816

[eva12394-bib-0025] Kelly, M. W. , E. Sanford , and R. K. Grosberg 2012 Limited potential for adaptation to climate change in a broadly distributed marine crustacean. Proceedings of the Royal Society of London B: Biological Sciences 279:349–356. 10.1098/rspb.2011.0542 PMC3223665 21653591

[eva12394-bib-0026] Kelly, M. W. , R. K. Grosberg , and E. Sanford 2013 Trade‐offs, geography, and limits to thermal adaptation in a tide pool copepod. The American Naturalist 181:846–854. 10.1086/670336 23669546

[eva12394-bib-0223] Kelly, M. W. , M. S. Pankey , M. B. DeBiasse , and D. C. Plachetzki 2016 Adaptation to heat stress reduces phenotypic and transcriptional plasticity in a marine copepod. Functional Ecology (accepted).

[eva12394-bib-0027] Kidder, G. W. , C. W. Petersen , and R. L. Preston 2006 Energetics of osmoregulation: II. Water flux and osmoregulatory work in the euryhaline fish, *Fundulus heteroclitus* . Journal of Experimental Zoology Part A: Comparative Experimental Biology 305:318–327, Wiley Online Library. 10.1002/jez.a.252 16493649

[eva12394-bib-0028] Latta, L. C. , L. J. Weider , J. K. Colbourne , and M. E. Pfrender 2012 The evolution of salinity tolerance in *Daphnia*: a functional genomics approach. Ecology Letters 15:794–802. 22583985 10.1111/j.1461-0248.2012.01799.x

[eva12394-bib-0029] Lau, J. A. , and C. P. terHorst 2015 Causes and consequences of failed adaptation to biological invasions: the role of ecological constraints. Molecular Ecology 24:987–1998. 10.1111/mec.13084 25677573

[eva12394-bib-0030] Law, C. W. , Y. Chen , W. Shi , and G. K. Smyth 2014 Voom: precision weights unlock linear model analysis tools for RNA‐seq read counts. Genome Biology 15:R29. 24485249 10.1186/gb-2014-15-2-r29 PMC4053721

[eva12394-bib-0031] Li, B. , and C. N. Dewey 2011 RSEM: accurate transcript quantification from RNA‐seq data with or without a reference genome. BMC Bioinformatics 12:323. 21816040 10.1186/1471-2105-12-323 PMC3163565

[eva12394-bib-0032] Oakley, C. G. , J. Ågren , R. A. Atchison , and D. W. Schemske 2014 QTL mapping of freezing tolerance: links to fitness and adaptive trade‐offs. Molecular Ecology 23:4304–4315. 25039860 10.1111/mec.12862

[eva12394-bib-0033] Pereira, R. J. , F. S. Barreto , and R. S. Burton 2014 Ecological novelty by hybridization: experimental evidence for increased thermal tolerance by transgressive segregation in *Tigriopus californicus* . Evolution 68:204–215. 24372605 10.1111/evo.12254

[eva12394-bib-0034] R Development Core Team 2014 R: A Language and Environment for Statistical Computing. R Foundation for Statistical Computing. doi: 10.1007/978‐3‐540‐74686‐7. http://www.r-project.org.

[eva12394-bib-0035] Ritchie, M. E. , B. Phipson , W. Di , H. Yifang , C. W. Law , W. Shi , and G. K. Smyth 2015 Limma powers differential expression analyses for RNA‐sequencing and microarray studies. Nucleic Acids Research gkv007:1–13: doi: 10.1093/nar/gkv007. 10.1093/nar/gkv007 PMC4402510 25605792

[eva12394-bib-0037] Schluter, D. 1996 Adaptive radiation along genetic lines of least resistance. Evolution 50:1766–1774, JSTOR. 10.1111/j.1558-5646.1996.tb03563.x 28565589

[eva12394-bib-0038] Schoville, S. D. , F. S. Barreto , G. W. Moy , A. Wolff , and R. S. Burton 2012 Investigating the molecular basis of local adaptation to thermal stress: population differences in gene expression across the transcriptome of the copepod *Tigriopus californicus* . BMC Evolutionary Biology 12:170. 22950661 10.1186/1471-2148-12-170 PMC3499277

[eva12394-bib-0040] Sokolova, I. M. 2013 Energy‐limited tolerance to stress as a conceptual framework to integrate the effects of multiple stressors. Integrative and Comparative Biology 53:597–608. 23615362 10.1093/icb/ict028

[eva12394-bib-0041] Sørensen, J. G. , T. N. Kristensen , V. Loeschcke , and M. F. Schou 2015 No trade‐off between high and low temperature tolerance in a winter acclimatized Danish *Drosophila subobscura* population. Journal of Insect Physiology 77:9–14. 25846012 10.1016/j.jinsphys.2015.03.014

[eva12394-bib-0042] Walsh, B. , and M. W. Blows 2009 Abundant genetic variation+ strong selection= multivariate genetic constraints: a geometric view of adaptation. Annual Review of Ecology, Evolution, and Systematics 40:41–59, Annual Reviews.

[eva12394-bib-0043] Willett, C. S. 2010 Potential fitness trade‐offs for thermal tolerance in the intertidal copepod *Tigriopus californicus* . Evolution 64:2521–2534. 20394668 10.1111/j.1558-5646.2010.01008.x

[eva12394-bib-0044] Williams, B. R. , B. Van Heerwaarden , D. K. Dowling , and C. M. Sgrò 2012 A multivariate test of evolutionary constraints for thermal tolerance in *Drosophila melanogaster* . Journal of Evolutionary Biology 25:1415–1426. 22587877 10.1111/j.1420-9101.2012.02536.x

